# Concentrations of Acute-Phase Proteins in Milk from Cows with Clinical Mastitis Caused by Different Pathogens

**DOI:** 10.3390/pathogens9090706

**Published:** 2020-08-27

**Authors:** Felipe M. Dalanezi, Elizabeth M. S. Schmidt, Sâmea F. Joaquim, Felipe F. Guimarães, Simoni T. Guerra, Bruna C. Lopes, Ronaldo L. A. Cerri, Christopher Chadwick, Hélio Langoni

**Affiliations:** 1 Department of Animal Production and Preventive Veterinary Medicine, Faculty of Medicine Veterinary and Animal Science, São Paulo State University (UNESP), São Paulo 18168-681, Brazil; felipe.dalanezi@unesp.br (F.M.D.); samea.joaquim@unesp.br (S.F.J.); felipefreitasguimaraes@hotmail.com (F.F.G.); simony.guerra@unesp.br (S.T.G.); bruna.c.lopes@unesp.br (B.C.L.); 2 Department of Veterinary Clinical Science, Faculty of Medicine Veterinary and Animal Science, São Paulo State University (UNESP), São Paulo 18168-681, Brazil; elizabeth.schmidt@unesp.br; 3 Applied Animal Biology, Faculty of Land and Food Systems, University of British Columbia, Vancouver, BC V6T 1Z4, Canada; ronaldo.cerri@ubc.ca; 4 Life Diagnosis, Inc., West Chester, PA 19380, USA; chris@lifediagnostics.com

**Keywords:** mastitis, dairy ruminants, cattle, acute-phase proteins, SPARCL

## Abstract

Among the new diagnostic methods for mastitis detection under development, milk acute-phase proteins (APPs) are receiving special attention. The study aimed to compare the profile of milk APPs from cows with natural clinical mastitis caused by distinct pathogens. The concentrations of haptoglobin (Hp), serum amyloid A (SAA), alpha-1-acid glycoprotein (AGP), and C-reactive protein (CRP) were measured by Spatial Proximity Analyte Reagent Capture Luminescence (SPARCL). Each APP was compared across the pathogens causing mastitis. The APPs differed statistically (*p* < 0.05) among the pathogens causing udder infection. There were significant and positive correlations among the concentration profile, for each pathogen, in three of four APPs studied. It can be concluded that the pathogen causing mastitis could modify the profile of release of the APPs in milk. The profile of Hp, AGP, and CRP demonstrated significant correlation, indicating that the three APPs are suggested as biomarkers, in milk, for bovine mastitis.

## 1. Introduction

Mastitis presents a significant challenge to global milk production, being responsible for an annual cost of approximately two billion dollars to dairy farmers in the United States alone [1]. Major costs related to mastitis were (1) reduction of milk production (US$ 102/cow/year), (2) discarding of milk (US$ 24/cow/year), and (3) animal replacement (US$ 33/cow/year), bringing the whole cost of mastitis to around US$ 159/cow/year [1]. Additionally, mastitis has a great influence on milk microbiota that could lead to changes in the milk composition decreasing the quality of the milk and milk byproducts [2]. Milk microbiota altered by mastitis could be the origin of foodborne diseases that affect humans [3].

Mastitis is characterized by inflammation of the mammary gland. It has multiple etiologies, and its most frequent etiological agents are bacteria [4], which lead to an inflammatory reaction [5]. Mastitis cases are classified as clinical or subclinical. The clinical form is characterized by abnormal milk (i.e., presence of blood, color change, or lumps) production, possible mammary gland tissue alteration, or systemic symptoms [6,7], while the subclinical form involves the mobilization of inflammatory cells to the udder, increasing the somatic cell count (SCC), but without alterations in gland tissue or milk aspect [8]. Mastitis control is dependent on diagnosis, with tests such as the California Mastitis Test (CMT); which exploits the reaction between the DNA from inflammatory cells in the milk and specific reagents, leading to the formation of a gel, which has a consistency that allows subjective analysis of the quantity of cells in the milk sample [9,10]. Alternatively, the number of cells in milk can be analyzed quantitatively via the SCC test. Some factors, such as the degree of infection, lactation state, age, breed, and milk transport, could alter the SCC [4]. Another relevant diagnostic procedure is microbiological culture to isolate the pathogen causing the infection; thus, a milk sample should be incubated in culture medium and checked for colony growth [4].

Recent studies have demonstrated that acute-phase proteins (APPs) from cows with mastitis are reliable as diagnostic biomarkers [11,12,13]. In bovines, the most relevant APPs in serum are haptoglobin (Hp), serum amyloid A (SAA), lipopolysaccharide binding protein (LBP), and alpha-1-acid glycoprotein (AGP) [14]. Haptoglobin, SAA, and c-reactive protein (CRP) have been identified in milk, indicating their potential as biomarkers of mastitis [15,16]. Recently, AGP has been identified in milk and mammary tissue, as a potential biomarker of mammary inflammation; however, research is currently limited [14]. 

Some specifics roles played by APPs at the site of inflammation include binding Hp to iron (to prevent the use of iron by bacteria), binding hemoglobin (to avoid oxidative damage), and anti-inflammatory and bacteriostatic effects [14]; SAA—modulation of innate immune reaction (to mediate the attraction, migration, and adherence of monocytes and neutrophils), opsonization of Gram-positive and -negative pathogens, and cholesterol binding [14]; AGP—reducing apoptosis, increasing expression of anti-inflammatory cytokines by macrophages and regulation of epithelial damage [17,18,19]; and CRP—regulating immune system during infections, destroying infectious agents, minimizing tissue damage, and facilitating tissue repair and regeneration [20,21]. According to the literature [11,14] APPs are produced locally (mammary gland) during an inflammatory reaction and have showed antibacterial activity against bacteria (SAA-3), antioxidant activities (HP), and mucin secretion, which is related to the innate immunity against the invasion and establishment of pathogens.

APPs are produced mainly in the liver, but some extrahepatic sites, such as mammary gland, white blood cells, reproductive system, respiratory and digestive tract, and adipose tissue could produce or release these proteins [14]. Increases in Hp, SAA, CRP, and AGP release in mammary gland cells have been observed in cases of mastitis, indicating the local production of these APPs in response to bacterial infection [12,22]. In addition, this approach is considered more accurate, as APPs have fewer interfering factors than the SCC test [23].

The objective of this study was to measure APPs from milk samples from cows with clinical mastitis caused by different pathogens (*Staphylococcus aureus*, *Escherichia coli*, environmental *Streptococcus*, coagulase-negative *Staphylococcus* (CNS), *Mycoplasma* spp., *Enterococcus* spp., or *Klebsiella pneumoniae*). We determined correlations between the concentrations of APPs, to understand the role of these proteins in diagnosing clinical mastitis caused by different pathogens.

## 2. Material and Methods

The study was approved by the Ethics Committee on Animal Use (CEUA) guidelines of the School of Veterinary Medicine and Animal Science (FMVZ)—São Paulo State University (UNESP), Brazil (protocol number: 196/2016—CEUA).

### 2.1. Microbiological Culture

This study was conducted in five commercial dairy herds with average milk production of 30 kg/day/cow, SCC lower than 400 × 10^3^ sc/mL, program of mastitis control (with herd management software), and at least 200 lactating cows. The herd on one farm was kept in facilities with cross-ventilation, using carousel-type milking; the animals were milked three times a day. The other herds were milked in a herringbone parlor; cows were kept in free-stall barns equipped with sprinklers and fans for cooling, and milked twice a day and fed a total mixed ration (TMR) with water access ad libitum. Milk samples from cows with a natural occurrence of acute clinical mastitis were used for microbiological isolation. Samples were collected, by convenience, by trained staff at the time point of clinical mastitis diagnosis, probably between 6 and 12 h after the onset of infection, using the strip cup test (i.e., presence of blood, color change, or lumps) [24]. Individual samples were collected aseptically, following the antisepsis of the teats with a 1% iodine–alcohol solution, and transported under refrigeration (4–8 °C) to the laboratory. Samples were cultured in bovine blood agar (5%), and MacConkey agar, incubated under aerobic conditions at 37 °C for 72 h, and examined for microbial growth every 24 h. Strains of microorganisms were identified according to culture, morphologic, and biochemical characteristics [25]. After pathogens were identified, milk samples were stored at −20 °C until APP determination.

Detection of *Mycoplasma* spp. was previously carried out as part of the standardized culture protocol followed for bovine milk samples, with cell numbers established as follows: 0.05 mL of each sample was cultured in a Petri dish filled with solid Hayflick medium, as described by Whitford et al. [26]. Incubation was carried out in a CO_2_ incubator under a microaerobic atmosphere, with observations of microbial growth carried out for at least 15 days after incubation. The isolated *Mycoplasma* spp. was evaluated in a stereo microscope, through visualization of colonies displaying characteristic “fried-egg” shapes and the formation of smears, as described by Pretto et al. [27].

### 2.2. Pathogen Identification

Microorganisms of the *Streptococcus* genus isolated from milk samples were differentiated by three tests: (1) The CAMP test: CAMP factor produced by the organism acts synergistically with beta-hemolysin produced by *Staphylococcus aureus* in blood agar; (2) Esculin hydrolysis: The main purpose is to verify whether bacteria are capable of hydrolyzing esculin as a source of carbon; (3) Bile–esculin hydrolysis: This test is based on the ability of *Streptococcus* to hydrolyze esculin in the presence of bile (4%) [25].

Samples containing *Staphylococcus* were categorized by the coagulase test [28]. Coagulase-positive strains were subjected to the following biochemical tests to identify *Staph. aureus*: the fermentation of sugars (trehalose, maltose, and mannitol), resistance to polymyxin B (300 UI), and sensitivity to novobiocin (5 mg). 

Colonies grown in MacConkey medium were identified through conventional biochemical tests. In the case of growth of non-specified pathogens causing mastitis, characterization was carried out according to the genus found, as described by Quinn et al. (2011) [25].

### 2.3. Determination of the Concentrations of Acute-Phase Proteins

To determine the concentrations of APPs in milk, 133 samples from cows diagnosed with clinical mastitis caused by different pathogens were used. The pathogens included *Escherichia coli* (n = 24), coagulase-negative *Staphylococcus* (CNS) (n = 24), *Mycoplasma* spp. (n = 18), *Enterococcus* spp. (n = 18), *Klebsiella pneumoniae* (n = 18), environmental *Streptococcus* (n = 16), and *Staphylococcus aureus* (n = 15). The concentrations of APPs (Hp, SAA, AGP, and CRP) were determined by the Spatial Proximity Analyte Reagent Capture Luminescence (SPARCL) assay, which uses two particular antibodies: one conjugated to a chemiluminescent substrate (acridan) and the other one to horseradish peroxidase (HRP). When HRP and acridan antibodies bind to their target, they approximate each other. Thus, by adding hydrogen peroxide, HRP catalyzes the oxidation of acridan, initiating a flash of chemiluminescence related to the biomarker concentration. Acridan molecules that are not close to HRP are not oxidized and do not generate luminescence. We used commercial kits to carry out the technique. For each APP assayed for, specific kits were employed: for Hp the Cow Haptoglobin Microplate SPARCL Assay (HAPT-SP-11), for SAA the Cow SAA Microplate SPARCL Assay (SAA-SP-11), for AGP the Cow AGP Microplate SPARCL Assay (AGP-SP-11), and for CRP the Cow CRP Microplate SPARCL Assay (CRP-SP-11). All kits were provided by Life Diagnostics Inc., West Chester, PA USA. Procedures were carried out according to the specification of the manufacturer. A specific luminometer (BMG LUMIstar Omega), set at a gain of 3600, was used for injection and simultaneous measurement of luminescence. In order to avoid a matrix effect, we found it necessary to dilute milk at least 16-fold with diluent for CRP, Hp, and SAA, and at least 100-fold for AGP with 50 μL of diluted milk samples.

### 2.4. SPARCL Immunoassay Milk Validation

To validate cow microplate Hp, SAA, AGP, and CRP SPARCL immunoassays, the following were used: Cow Haptoglobin antigen, KLH conjugated SAA3 peptides (two) antigen, alpha-1 acid glycoprotein antigen, and Cow CRP antigen. Standard calibrators were: Cow Haptoglobin, Cow SAA3, Cow *α1* acid glycoprotein, and Cow CRP.

The precision was determined as the mean coefficient of variance (CV) with triplicate assay for four samples (n = 12) with varying concentrations of the biomarker repeated in a single assay for intra-assay and over repeated assays for inter-assay CV. The accuracy was determined by parallel dilution of samples with high concentrations of the biomarker; the mean (±SD) of the observations was divided by the expected concentration and expressed as a percentage. The minimal detected concentration of the assays was determined as the lowest concentration of purified protein that could be measured in buffer and in milk after adjustment for the minimum dilution required to eliminate matrix effects. Specificity was dependent on the nature of the antigen employed to produce the antibodies used in the assays. 

The concentrations of Hp in milk samples were compared in cows with clinical mastitis caused by different pathogens, determined by Enzyme-Linked Immunosorbent Assay (ELISA) and SPARCL. For the comparison between the methods, the same milk samples were used in both analyses. The ELISA assay uses two antibodies to detect a ligand, usually a protein; the first antibody is attached to the plate and the second is linked to the protein. For ELISA analysis, the Haptoglobin ELISA (Life Diagnostics Inc., West Chester, PA, USA) was used, procedures were carried out according to the specification of the manufacturer.

### 2.5. Statistical Analysis 

Data were first analyzed for normality of distribution using the Kolmogorov–Smirnov and Shapiro–Wilk tests. As the data were not normally distributed, a non-parametric (Wilcoxon test) comparison among different pathogens and APPs, and Dunn’s post hoc analysis (using Bonferroni error correction to adjust for multiple comparisons) were performed. A non-parametric (Spearman’s correlation) analysis was employed to check the correlations among the Hp, SAA, AGP, and CRP concentrations and correlation between the different methods of Hp mensuration, ELISA and SPARCL. *p*-values were considered significant at *p* < 0.05. All tests were performed using the software SAS University^®^ (SAS Institute Inc., Cary, NC, USA).

## 3. Results

Intra- and inter-assay coefficients of variation (CVs) were below 8% and 15%, respectively, for all the assays evaluated, except for AGP (inter-assay 30.2%) (Table 1).

Overall, the analytical validation tests showed that the assays used in our study are valid and reliable for the evaluation of acute-phase proteins in milk by the SPARCL method. The Spearman correlation analysis demonstrates a significant positive correlation (r_s_ = 0.80, *p* < 0.001) between the two methods (ELISA and SPARCL).

Some drawbacks of the method are the necessity of a specific luminometer and the adjustment of the dilution of the samples to fit the measurement within the detection range of the assay. Besides that, since the commercial kits provide plates and reagents, the technique is similar to ELISA but easier. The only drawback that could affect the measurement of the APPs was observed in the case of SAA, where the isoform present in the kit may not have detected the protein accurately. However, this is only speculation, and more studies must be performed to prove the hypothesis.

Cows enrolled in the study were on average in the fourth lactation, and the percentage of quarters infected was: front right—24.8% (33/133), front left—32.3% (43/133), rear right—24.8% (33/133), and rear left—18.1% (24/133). The control group (n = 8), non-infected cows with SCC lower than 200 × 10^3^ cells/mL, had a concentration range (µg/mL) for Hp of 0.0, SAA from 0.0 to 8.7; AGP from 6.1 to 8.8, and CRP from 0.07 to 0.13.

Concentrations of milk Hp (Table 2) were significantly higher (*p* < 0.05) in samples from mastitis caused by *E. coli*, *K. pneumoniae, Staph. aureus*, or environmental *Streptococcus,* and significantly lower (*p* < 0.05) in mastitis samples attributable to *Enterococcus* spp. or CNS. Samples from mastitis caused by *Mycoplasma* spp. had moderate concentrations of Hp when compared to that for all other pathogens.

Milk SAA concentrations (Table 2) were significantly higher (*p* < 0.05) in samples from cows infected with environmental *Streptococcus* than that in those infected with *K. pneumoniae* or *Enterococcus* spp. Milk samples from animals infected with *Staph. aureus* or *Mycoplasma* spp. had concentrations similar to those with *K. pneumoniae* or *Enterococcus* spp. infections, but different from those with environmental *Streptococcus*. Significantly lower SAA (*p* < 0.05) concentrations were observed in samples from animals with *E. coli* or CNS infections.

Concentrations of milk AGP (Table 2) were significantly higher (*p* < 0.05) in samples from mastitis caused by *E. coli* or *Staph. aureus*, in relation to those caused by *K. pneumoniae*. Samples from mastitis caused by environmental *Streptococcus* or *Mycoplasma* spp. presented concentrations similar to that of those with *K. pneumoniae* infection, but different from those with *E. coli* or *Staph. aureus.* Milk samples from mastitis caused by *Enterococcus* spp. or CNS had significantly lower (*p* < 0.05) concentrations when compared to that caused by the other pathogens.

Milk CRP (Table 2) concentrations were significantly higher (*p* < 0.05) in samples from cows with *E. coli* or *K. pneumoniae* infections; moderate for mastitis caused by *Staph. aureus,* environmental *Streptococcus,* and *Mycoplasma* spp.; and significantly lower (*p* < 0.05) in mastitis caused by *Enterococcus* spp. or CNS.

Significant positive correlations (Table 3) were observed between Hp and SAA (r_s_ = 0.39, *p* < 0.001), Hp and AGP (r_s_ = 0.60, *p* < 0.001), Hp and CRP (r_s_ = 0.55, *p* < 0.001), and AGP and CRP (r_s_ = 0.72, *p* < 0.001).No significant correlation was observed between SAA and AGP (r_s_ = 0.11, *p* = 0.18), or between SAA and CRP (r_s_ = 0.05, *p* = 0.55).

## 4. Discussion

To the best of the authors’ knowledge, this is the first study reporting the use of the SPARCL assay to determine milk APP concentrations in cows with clinical mastitis. Additionally, this is the first time that AGP was detected in milk samples from cows with clinical mastitis caused by different pathogens. The results of this study demonstrated differences between the concentrations of Hp, SAA, CRP, and AGP in milk derived from clinical infection by different pathogens. 

Cows infected with *E. coli*, *K. pneumoniae*, or environmental *Streptococcus* had higher concentrations of Hp compared to those infected by other pathogens. This result has previously been reported for *E. coli* infection and reflects the marked response of the udder cells to bacterial damage [12,29,30]. This is the first study reporting the detection of Hp in milk from mastitis caused by *K. pneumoniae*. The increased concentration of this APP was expected because, similarly to *E. coli*, this microorganism causes acute clinical mastitis [31]. The lipopolysaccharide (LPS) present in the membrane of *E. coli* and *K. pneumoniae* modulates the production of APPs by stimulating the synthesis of tumor necrosis factor-α (TNF-α). Thus, *E. coli* and *K. pneumoniae* could have the same pattern in the development of clinical mastitis because both have similar LPS molecules in their bacterial membranes. However, *K. pneumoniae* usually causes more severe mastitis than *E. coli* due to a small difference in one of the constitutive molecules of LPS [31], as observed in the present study by the concentration of milk Hp. In the acute-phase reaction, along with the increase in TNF-α there is an augmentation in the migration of polymorphonuclear cells (PMNs) to the mammary gland, leading to a higher number of phagocytic cells, thus increasing the killed bacteria, which releases more LPS, thereby stimulating the inflammatory response and APP production [32]. Additionally, migration of PMNs to the udder could be stimulated by Hp since one role of this protein is the recruitment of neutrophils [33]. The higher concentration of Hp in milk infected by environmental *Streptococcus* was also expected, as previous studies have demonstrated high concentration of this APP in mastitis caused by this pathogen [12,34]. The high concentration of Hp, in milk samples from cows with mastitis caused by environmental *Streptococcus*, may be related to a notable influx of PMNs after streptococcal infection, increasing the release of Hp in milk [35].

Gram-positive bacteria are well known for causing chronic mastitis instead of acute mastitis [36] because of the lack of LPS [37], although *Streptococcus uberis* (an environmental *Streptococcus*) could lead to a pronounced inflammatory response in mammary gland infections resulting in an increase of APPs such as Hp [35]. Gram-positive pathogens require more hours after infection to increase cytokines and APPs, but the higher concentration persists for a longer period when compared to Gram-negative bacteria [35,36,38,39]. Thus, the unknown period between the onset of infection and collection of the milk samples could explain the similar Hp concentrations among *Streptococcus*-infected cows and coliform-infected cows. *Escherichia coli, K. pneumoniae*, and environmental *Streptococcus* are known to release reactive oxygen species (ROS) [40] by triggering Hp production and decreasing the concentration of ROS in those pathogenic infections [14].

The udder infections caused by *Mycoplasma* spp. or *Staph. aureus* moderately increased the concentrations of milk Hp. The concentrations of this APP were similar among *Staph. aureus*, *E. coli*, *K. pneumoniae,* and environmental *Streptococcus*. Mastitis caused by *Mycoplasma* spp. increased the Hp concentration similarly to that caused by *Staph. aureus*. Although a low concentration of Hp in milk samples from cows with an *Staph. aureus* infection has been previously reported [12], the moderate Hp concentration in *Staph. aureus* infection in our study was similar to that reported by Kalmus et al. [30]. 

This is the first study to report the concentration of Hp in mastitic milk from *Mycoplasma* spp. infection. This pathogen presents a behavior analogous to *Staph. aureus*-infection, resulting in chronic and persistent disease [41]. *Mycoplasma* spp. has a similar pattern of immune system stimulation as *Staph. aureus*, and both pathogens have a low growth rate in the mammary gland [42]. The reduced growth of these pathogens could lead to a small increase in Hp concentration as observed in the present study. A minor inflammatory response is expected from infection by these bacteria, causing a moderate stimulus for Hp production.

This is the first time that Hp was detected in milk from mastitis caused by *Enterococcus* spp., demonstrating that this microorganism elicits a similar response to that of CNS. Previous investigations reported that the production of a biofilm may be related to the pathogenicity of *Enterococcus* species, which helps the bacterium to invade host cells and to avoid the immune response, thus decreasing the local inflammation and resulting in a lower production of APPs [43,44]. Mastitis caused by either *Enterococcus* spp. or CNS resulted in lower Hp concentrations, suggesting that these bacteria promoted a mild inflammatory response in the udder cells, as reported previously [45]. The Hp concentration in mastitic milk from CNS infection was consistent with the literature when compared to that from infections by other pathogens [29,30]. 

The cows with mastitis caused by environmental *Streptococcus* had the highest SAA concentration in milk. Udder infection by *K. pneumoniae* and *Enterococcus* spp. released an SAA concentration lower than that of environmental *Streptococcus* and higher than that of *Mycoplasma* spp. and *Staph. aureus*, thereby demonstrating a moderate concentration of SAA in milk samples infected by *K. pneumonia* or *Enterococcus* spp. Both SAA and Hp were increased in acute cases when compared to chronic cases [20], a finding that corroborates the results found in the present study, since *Mycoplasma* spp. and *Staph. aureus* cause chronic mastitis [41]. In *Staph. aureus*-infected samples the SAA concentration was similar to those reported in previous studies [29,30], indicating that this detection is plausible and could explain the concentrations reported for the other bacteria. Since one of the roles of SAA at an inflammatory site is to modulate the immune reaction [14], it was expected that pathogens causing more acute mastitis such as *K. pneumoniae* and environmental *Streptococcus* would lead to high concentrations of these proteins in milk. Similarly, pathogens that cause chronic and longer infection as *Mycoplasma* spp. and *Staph. aureus* provoked a less pronounced immune reaction and consequently lower concentration of SAA. *Enterococcus* spp. led to a high concentration of SAA, which may be attributable to a different mechanism of stimulating the production and release of the protein, or an artefact of the method since the other APPs presented low concentrations in milk samples infected by this pathogen. More studies need to be done to account for this result.

The low concentration of SAA in samples from CNS infection was expected, as this bacterium is not related to severe inflammation in the udder cells. Therefore, SAA production was not detected. Elevated SAA concentrations were reported in milk samples from mastitis caused by *E. coli* [12,29,30], whereas in our study the SAA concentrations were diminished. These results may reflect the fact that a different method was utilized to detect the APPs, whereas a milk-specific SAA method would be necessary. A higher SAA concentration was expected in *E. coli* milk samples, similar to *K. pneumoniae* results. However, these two pathogens differ specifically in one LPS factor [31] that could stimulate the immune response differently, thus producing an isoform of SAA not fully detected by the assay. Samples from CNS-infected cows had low SAA concentrations in accordance with previous studies [29,30,46], as CNS usually causes mild clinical or subclinical mastitis with minor stimulation of the immune system, lower production of cytokines, and consequently, low APPs. 

It has been demonstrated that AGP is produced by alveolar cells of the mammary gland, but the major part of AGP in milk is released by white blood cells [17]. The same authors reported that AGP increases drastically during mammary infection. Comparison of AGP concentrations in milk from cows with minor or moderate clinical mastitis versus those without mastitis demonstrated significant differences between cows with subclinical mastitis and healthy cows [47], but there was no difference between cows with minor versus moderate clinical mastitis [48]. Alpha-1 glycoprotein concentrations in milk from cows with subclinical mastitis caused by *E. coli*, *Streptococcus* spp., or *Staphylococcus* spp., were higher than those in non-mastitic cows, but AGP did not differ among these three bacteria [47]. In our study, there were significant differences among milk AGP concentrations from cows with mastitis caused by different pathogens. Mastitis caused by *E. coli* or *Staph. aureus* presented higher AGP concentrations. A hypothesis explaining why AGP was higher in milk samples from cows with *E. coli* or *Staph. aureus* would be that the udder response in cows challenged with LPS increased the AGP concentration [49]. A similar pattern was expected for the AGP concentration in milk samples with *K. pneumoniae* infections given that the concentration of this APP was elevated in mastitis caused by this pathogen [50]. Reducing apoptosis is one of the roles of AGP at the inflammatory site; therefore, it was demonstrated that mammary infections by *E. coli* or *Staph. aureus* lead to apoptosis of epithelial cells of the udder [51], and thus could stimulate the production of AGP. The LPS molecule increases the production of cytokines [52]. One of the roles of the AGP is to elevate the production of these cytokines [19], enabling the proteins to elicit the increase of AGP in mastitis caused by Gram-negative pathogens. Mammary infection by *Staph. aureus* does not increase the production and release of TNF-α as does infection by Gram-negative bacteria, but it was demonstrated that *Staph. aureus* could increase the concentration of other cytokines that could lead to an elevated APP concentration [53]. Gram-negative bacteria are related to a marked APP response, while Gram-positive bacteria induce a mild response [36], so a moderate concentration of the APP in mastitis caused by *Streptococcus* was expected, as was observed in our study. The data on AGP concentration in mastitis caused by *Mycoplasma* spp. are scarce, but the results indicate that this pathogen is capable of inducing a moderate increase of AGP concentration as observed for the other three APPs. Milk samples from cows infected with *Enterococcus* spp. or CNS had lower AGP concentrations, suggesting the mild effect of these pathogens on the synthesis and release of this APP. Gram-positive bacteria usually trigger mild clinical cases of mastitis inducing scarce mobilization of immune cells [36]. A high AGP concentration is associated with great mobilization of white blood cells [17], so a mild mobilization of these cells to the udder could be related to a low concentration of the protein. Corroborating that, it was demonstrated that white blood cells could stimulate the apoptosis of epithelial cells of the udder [51], provoking an increase in AGP concentration, so pathogens that cause more pronounced influx of white cells could lead to greater APP concentration.

Milk CRP concentrations in the cows of our study were similar to previous reports of milk CRP values from cows with mastitis caused by different pathogens [12,54]. Mastitis caused by Gram-negative bacteria is known for its severe clinical cases that produce tissue damage [52], which stimulates CRP activation [20], as previously reported in *E. coli* infection [12]. In agreement with our study, an increase of CRP concentration was observed in milk samples from cows with mastitis caused by *E. coli*. Mammary infections by *Staph. aureus*, environmental *Streptococcus* or *Mycoplasma* spp. produced a moderate CRP milk concentration. Thomas et al. [12] reported a high CRP concentration in milk samples infected with *Strep. uberis* or *Strep. dysgalactiae* (environmental *Streptococcus*), in contrast to the results obtained in the present study. The results of that same previous study of CRP concentrations of samples infected with *Staph. aureus* were similar to those of our study, indicating moderate damage caused by this pathogen to the udder cells. Gram-positive bacteria cause mild and chronic cases of mastitis in cows, a type of infection that leads to milder tissue damage [36]. This moderate tissue damage could stimulate CRP production but without elevating its concentration as high as that caused by Gram-negative bacteria. Concentration of CRP in milk from mastitis caused by *Mycoplasma* spp. is not well described in the literature; in all APPs studied in this article the pathogens demonstrated a moderate pattern of stimulating APP production, indicating a non-severe inflammatory response in mastitis caused by *Mycoplasma* spp. Mammary infection with CNS or *Enterococcus* spp. showed a lower CRP concentration in comparison to that caused by the aforementioned bacterium. The detection of CRP in milk from CNS infection was in accordance with the literature [12], indicating that *Enterococcus* spp. could induce a mild inflammatory response in the mammary gland, similar to that of CNS. The mild inflammatory response could be linked to the mild tissue damage observed in mastitis caused by CNS infections [55].

There was a strongly positive significant correlation between milk Hp, AGP, and CRP concentrations from cows with mastitis, which could indicate that pathogens with similar concentration patterns for these APPs induced similar inflammatory responses in the epithelial cells of the udder. No significant correlation was found between SAA, CRP, and AGP, indicating that these APPs of different mastitis pathogens are not related and do not cause a similar inflammatory reaction. These data support previous reports in the literature, which found a moderate correlation between Hp and SAA (r_s_ = 0.39) [29] and also a strong correlation between Hp and M-SAA3 [12]. In agreement with these same authors, the results of this study revealed a moderate correlation between Hp and CRP (r_s_ = 0.55). The strong correlations between AGP and Hp (r_s_ = 0.60) and between AGP and CRP (r_s_ = 0.72), indicate that AGP might be a good biomarker for mastitis.

New tools for mastitis diagnostics are being studied to achieve a more accurate and faster detection of udder infection, among these tools are the milk proteins. Milk proteomics has been analyzed by researchers to find new biomarkers for mastitis diagnostic [56]. The APPs are one type of milk protein that could be used for mastitis diagnosis. This article provides some information about four APPs that could, in the future, be used as mastitis biomarkers in milk.

## 5. Conclusions

Overall, this is the first report of milk APPs in mastitis caused by *K. pneumoniae*, *Mycoplasma* spp., or *Enterococcus* spp. A similar pattern of APP concentrations was observed in *K. pneumoniae* and *E. coli* samples. Additionally, *Mycoplasma* spp. and *Staph. aureus* shared a similar milk APP concentration pattern, whereas the same profile of APP concentrations was detected in *Enterococcus* spp. and CNS milk samples. The study reinforces that Hp, AGP, and CRP could be used as tools for diagnosing bovine mastitis caused by different pathogens, as already demonstrated by other authors.

## Figures and Tables

**Table 1:** Mean, standard deviation (SD), and intra- and inter-assay coefficients of variation (CVs) in each analyte concentration of four milk samples of cows.

	Precision		Accuracy: Parallel Dilutions	Detection Limit (ng/mL)	
	Intra-Assay CV	Inter-Assay CV	Mean (+SD) of O/E (%)	In Buffer	In Milk
**α-1 acid glycoprotein**	5.3	30.2	104.4 ± 11.9	1.95	97.5
**C-reactive protein**	2.7	7.2	81.1 ± 14.1	0.78	39
**Haptoglobin**	6.2	10.7	100.3 ± 3.4	1.95	97.5
**Milk amyloid-A (SAA3)**	3.0	5.7	96.0 ± 5.3	0.47	23.5

**Table 2:** Concentration of acute-phase proteins (APPs) (µg/mL) in milk of cows with clinical mastitis caused by different pathogens.

	Haptoglobin	Serum Amyloid A	α1-Acid Glycoprotein	C-Reactive Protein
***Klebsiellapneumoniae* (N = 18)**	206.1 ^A^ (0.0–1113.3) [126.3–468.1]	52.4 ^A,B^ (2.0–178.8) [31.9–97.1]	34.9 ^A,B^ (14.4–305.0) [22.4–145.3]	1.6 ^A^ (0.1–8.0) [0.5–4.4]
***Escherichia coli* (N = 24)**	164.1 ^A^ (0.0–2009.4) [71.7–305.1]	20.5 ^C^ (0.0–264.0) [8.6–47.2]	47.3 ^A^ (6.8–892.9) [24.8–155.7]	2.0 ^A^ (0.1–7.8) [0.8–4.8]
***Staphylococcus aureus* (N = 15)**	158.7 ^A,B^ (0.0–596.1) [0.0–300.0]	38.2 ^B^ (13.3–129.5) [21.8–94.9]	51.3 ^A^ (11.9–289.9) [17.3–207.4]	0.8 ^B^ (0.0–9.2) [0.1–2.0]
**Environmental *Streptococcus* (N = 16)**	179.0 ^A^ (0.0–812.2) [130.2–363.7]	63.8 ^A^ (21.0–151.4) [48.4–70.6]	29.7 ^B^ (11.3–100.7) [19.0–49.3]	0.6 ^B^ (0.1–5.4) [0.3–1.4]
***Mycoplasma* spp. (N = 18)**	102.0 ^B^ (0.0–582.9) [0.0–332.8]	35.2 ^B^(0.0–102.2) [17.4–51.5]	24.7 ^B^ (10.6–427.5) [15.1–79.2]	0.6 ^B^ (0.1–6.8) [0.3–2.1]
***Enterococcus* spp. (N = 18)**	43.0 ^C^ (0.0–213.0) [0.0–127.3]	55.4 ^A,B^(0.0–250.0) [26.8–86.1]	17.9 ^C^ (10.1–297.5) [15.8–22.2]	0.2 ^C^ (0.1–2.6) [0.1–0.7]
**CNS (N = 24)**	0.0 ^C^ (0.0–319.1) [0.0–66.3]	14.9 ^C^ (0.0–141.7) [8.7–37.7]	9.7 ^C^ (5.9–42.6) [7.8–9.7]	0.1 ^C^ (0.1–3.5) [0.1–0.4]

Data with distinct letters (^A,B,C^) in the rows are significantly different (*p* < 0.05). Hp—haptoglobin; SAA—serum amyloid A; AGP—alpha-1-acid glycoprotein; CRP—C-reactive protein; CNS—coagulase-negative *Staphylococcus*. The values indicate the median, (minimum–maximum), and [Q1–Q3].

**Table 3:** Correlations between different milk APPs of cows with clinical mastitis (r_s_ values).

APPs	Hp	SAA	AGP	CRP
**Hp**	1.00			
**SAA**	0.39 *	1.00		
**AGP**	0.60 *	0.11	1.00	
**CRP**	0.55 *	0.05	0.72 *	1.00

* Significantly different (*p* < 0.05). Hp—haptoglobin; SAA—serum amyloid A; AGP—alpha-1-acid glycoprotein; CRP—C-reactive protein.
